# Mutation update: Review of *TPP1* gene variants associated with neuronal ceroid lipofuscinosis CLN2 disease

**DOI:** 10.1002/humu.23860

**Published:** 2019-07-26

**Authors:** Emily Gardner, Mitch Bailey, Angela Schulz, Mikel Aristorena, Nicole Miller, Sara E. Mole

**Affiliations:** ^1^ UCL MRC Laboratory for Molecular Cell Biology and UCL Great Ormond Street Institute of Child Health University College London London United Kingdom; ^2^ Global Scientific Affairs BioMarin Pharmaceutical Inc Novato California; ^3^ Department of Paediatrics University Medical Center Hamburg‐Eppendorf Hamburg Germany

**Keywords:** genotype–phenotype correlation, late‐infantile neuronal ceroid lipofuscinosis, lysosomal storage disorders, neurodegeneration, tripeptidyl peptidase I

## Abstract

Neuronal ceroid lipofuscinosis type 2 (CLN2 disease) is an autosomal recessive condition caused by variants in the *TPP1* gene, leading to deficient activity of the lysosomal enzyme tripeptidyl peptidase I (TPP1). We update on the spectrum of *TPP1* variants associated with CLN2 disease, comprising 131 unique variants from 389 individuals (717 alleles) collected from the literature review, public databases, and laboratory communications. Previously unrecorded individuals were added to the UCL *TPP1*‐specific database. Two known pathogenic variants, c.509–1 G>C and c.622 C>T (p.(Arg208*)), collectively occur in 60% of affected individuals in the sample, and account for 50% of disease‐associated alleles. At least 86 variants (66%) are private to single families. Homozygosity occurs in 45% of individuals where both alleles are known (87% of reported individuals). Atypical CLN2 disease, TPP1 enzyme deficiency with disease onset and/or progression distinct from classic late‐infantile CLN2, represents 13% of individuals recorded with associated phenotype. NCBI ClinVar currently holds records for 37% of variants collected here. Effective CLN2 disease management requires early diagnosis; however, irreversible neurodegeneration occurs before a diagnosis is typically reached at age 5. Timely classification and public reporting of *TPP1* variants is essential as molecular testing increases in use as a first‐line diagnostic test for pediatric‐onset neurological disease.

## Graphical abstract

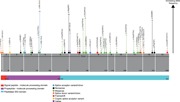

## 1 INTRODUCTION

The neuronal ceroid lipofuscinoses (NCLs) are a heterogeneous group of neurodegenerative lysosomal storage disorders characterized by the accumulation of neuronal and extraneuronal ceroid lipopigments (Jalanko & Braulke, 2009). To date, mutations in 13 human genes have been linked with NCL disorders (Mole, 2017). Classic late‐infantile neuronal ceroid lipofuscinosis, CLN2 disease, is the result of tripeptidyl peptidase I (TPP1) deficiency, caused by autosomal recessive inheritance of two pathogenic variants in trans in the *TPP1* (MIM# 607998, *CLN2)* gene (Fietz et al., 2016; Mole, Gardner, Schulz, & Xin, 2018; Sleat et al., 1997).

CLN2 disease (MIM# 204500) classically presents with seizure onset at 2–4 years of age, preceded by delayed language development, and followed by rapidly progressing dementia, psychomotor decline (loss of the ability to walk and talk), epilepsy, blindness, and death, typically between 6 years of age and the early teenage years (Mole et al., 2018; Mole, 2001; Nickel et al., 2016; Nickel et al., 2018; Steinfeld et al., 2002). Whereas classic late‐infantile CLN2 disease has a very well defined natural history, there exists a phenotypic spectrum of TPP1 enzyme deficiency in small numbers of patients, some with later onset or protracted disease course (Kohan et al., 2013; Kousi, Lehesjoki, & Mole, 2012). One form of juvenile onset disease was initially described as spinocerebellar ataxia 7 (SCAR7; MIM# 609270) and was later attributed to a TPP1 enzyme deficiency (Sun et al., 2013). Other, variant forms of complex hereditary spastic paraplegia (Kara et al., 2016) and childhood‐onset progressive ataxia (Dy, Sims, & Friedman, 2015) were described clinically before being linked to TPP1 enzyme deficiency. Occasional cases present before the age of 2 years (Nickel et al., 2018). With the knowledge of a shared molecular etiology, rather than being distinct entities, these diseases can be considered part of the same phenotypic spectrum that includes classic late‐infantile CLN2 disease and forms of atypical CLN2 disease. Thus, NCL classification was revised to take into account such phenotypic variation (Williams & Mole, 2012).

Effective CLN2 disease management requires timely diagnosis; however, irreversible neurodegeneration often occurs before a diagnosis is typically reached at 5 years of age (Nickel et al., 2018). Early diagnosis has become even more relevant as a recently approved intracerebroventricular enzyme replacement therapy has been shown to effectively slow the rapid decline in motor and language function in patients with CLN2 disease (Schulz et al., 2018). Aside from genetic testing, there are other medical procedures that may increase suspicion of CLN2 disease, for example, severe cerebellar atrophy is the principal sign seen at the time of diagnosis on magnetic resonance imaging (MRI) (Williams et al., 2006). Photosensitivity, as detected by electroencephalography, is also an early marker of CLN2 disease (Specchio et al., 2017).

The American College of Medical Genetics (ACMG) guidelines recommend that gene variants be reported in combination with their assessed pathogenicity (Richards et al., 2015). The purpose of this mutation update is to summarize the identified disease‐related genetic variation in the *TPP1* gene, with emphasis on clinical classification and genotype–phenotype correlations. There is a clear set of patients with atypical CLN2 disease which includes TPP1 deficiency, from SCAR7 and juvenile NCL. We collected and analyzed variant information from 389 individuals (131 different/independent variants) associated with CLN2 disease to uniformly summarize all *TPP1* gene variants.

## 2 METHODS

### 2.1 Data sources

Data from the University College London (UCL) *TPP1* Locus‐specific Database (https://www.ucl.ac.uk/ncl-disease/) was combined with the described literature searches to collect all *TPP1* variants reported to be associated with TPP1 enzyme deficiency and/or related disorders. A PubMed literature search was performed on May 22, 2018, using the following terms:

((CLN2[title] OR Tripeptidyl peptidase[title]) OR (Batten[title] OR NCL[title] OR neuronal ceroid lipofuscinosis[title]) AND (late infantile[title] OR late‐infantile[title])) AND ((“mutation”[mesh terms] OR “mutation”[all fields]) OR (“genotype”[mesh terms] OR “genotype”[all fields]) OR (“variant”[mesh terms] OR “variant”[all fields])).

Embase was searched using the following searches:

1. (‘tpp1 gene’/de OR ‘cln2 gene’/de OR ‘tripeptidyl peptidase i’/de OR ‘e.c. 3.4.14.9’ OR ‘tripeptidyl peptidase 1′ OR ‘tripeptidyl peptidase i’ OR 'tripeptidyl peptide hydrolase i’ OR ‘tripeptidylpeptidase 1’ OR ‘tripeptidylpeptidase i’ OR 'tripeptidylpeptide hydrolase i’) AND (‘mutation’/de OR ‘gene alteration’ OR ‘genome mutation’ OR ‘mutation’) AND (‘human’/de)
2. 'neuronal ceroid lipofuscinosis’/de AND ‘mutation’ AND ‘cln2’ AND ‘human’/de NOT ((‘tpp1 gene’/de OR ‘cln2 gene'/de OR ‘tripeptidyl peptidase i’/de OR ‘e.c. 3.4.14.9’ OR ‘tripeptidyl peptidase 1’ OR ‘tripeptidyl peptidase i’ OR ‘tripeptidyl peptide hydrolase i’ OR ‘tripeptidylpeptidase 1’ OR ‘tripeptidylpeptidase i’ OR ‘tripeptidylpeptide hydrolase i’) AND (‘mutation’/de OR 'gene alteration’ OR ‘genome mutation’ OR 'mutation’) AND ‘human’/de).

All variants collected from the UCL *TPP1*‐specific database and literature searches were assessed using ACMG standards and guidelines for interpretation of sequence variants using available information (Richards et al., 2015). Variants collected from the literature were compared to, and combined with, pathogenic or likely pathogenic variants contained in ClinVitae, a database including the National Center for Biotechnology Information (NCBI)’s ClinVar, in addition to several diagnostic laboratories (http://clinvitae.invitae.com/; accessed March 8, 2018). Variant‐level summaries include variants from all sources, whereas individual‐level summaries include literature and database cases, where such information was available.

Visualization of TPP1 was created using The PyMOL Molecular Graphics System, Version 1.8 Schrödinger, LLC with atomic coordinates from Brookhaven Protein Data Bank accession number 3EE6 (Pal et al., 2009).

### 2.2 Mutation nomenclature

The mutation nomenclature used in this update follows the guidelines indicated by the Human Genome Variation Society (den Dunnen et al., 2016). For the description of sequence variants, we used reference sequence NM_000391.3 for *TPP1* gene. Nucleotide numbering reflects cDNA numbering with position +1 corresponding to the A of the ATG translation initiation codon at nucleotide 62. Mutation descriptions on the protein level consider the initiator methionine as codon 1.

## 3 RESULTS

### 3.1 *TPP1* mutation spectrum

166 publications were returned using the three searches; eight were unavailable to review. Of the 158 publications reviewed, 90 contained *TPP1* variants. All new reports were added to the locus‐specific database at UCL.

Overall, 717 alleles were collected from 389 individuals reported in the literature and/or the UCL database, resulting in 131 different/independent *TPP1* variants (Table 1). It should be noted that the effect on TPP1 function has not been established for all disease‐associated alleles. In some patients, further variant alleles were found, in addition to those presumed to be disease‐associated. Four of these additional alleles are described in the UCL *TPP1* Locus‐specific Database.

**Table 1:** *TPP1* variants expected to cause associated TPP1 enzyme deficiency

Location	Nucleotide change	Amino acid change	# Alleles	ClinVar: Clinical significance	Contig position (GRCh38.p7)	Reference
Intron 01	c.17 + 1 G>C	NA	4	NA	6619383 C>G	Kousi et al. (2012)
Intron 01	c.18–3 C>G	NA	1	NA	6619270 G>C	Kousi et al. (2012)
Exon 02	c.37dup	p.(Leu13Profs*32)	1	NA	6619248dup	Kousi et al. (2012)
Exon 02	c.38 T>C	p.(Leu13Pro)	1	NA	6619247 A>G	M. Nickel, personal communication
Intron 02	c.89 + 1 G>A	NA	2	NA	6619195 C>T	Saini, Sankhyan, and Singhi (2016)
Intron 02	c.89 + 4 A>G	NA	2	NA	6619192 T>C	Noher de Halac et al. (2005)
Intron 02	c.89 + 5 G>C	NA	8	Pathogenic	6619191 C>G	Kousi et al. (2012)
Exon 03	c.139 C>G	p.(Leu47Val)	1	NA	6618866 G>C	E. de los Reyes, personal communication
Exon 03	c.177_180del	p.(Glu59Aspfs*20)	1	NA	6618825_6618828del	Chang et al. (2012)
Exon 03	c.184 T>A	p.(Ser62Thr)	1	NA	6618821 A>T	Kousi et al. (2012)
Exon 03	c.184_185del	p.(Ser62Glyfs*25)	1	NA	6618820_6618821del	Lam, Poon, Tong, and Ko (2001)
Exon 03	c.196 C>T	p.(Gln66*)	7	Pathogenic	6618809 G>A	Sleat et al. (1999)
Exon 03	c.225 A>G	p.( = )	5	NA	6618780 T>C	Sleat et al. (1999)
Exon 03	c.229 G>A	p.(Gly77Arg)	4	Pathogenic	6618776 C>T	Sleat et al. (1999)
Exon 03	c.229 G>T	p.(Gly77*)	1	NA	6618776 C>A	Chang et al. (2012)
Intron 03	c.229 + 3 G>C	NA	2	NA	6618773 C>G	R. Williams, personal communication
Exon 04	c.237 C>G	p.(Tyr79*)	1	NA	6617769 G>C	Kousi et al. (2012)
Exon 04	c.299 A>G	p.(Gln100Arg)	4	Benign/likely benign	6617707 T>C	Sleat et al. (1999)
Exon 04	c.311 T>A	p.(Leu104*)	4	Pathogenic	6617695 A>T	Kohan et al. (2008)
Exon 04	c.337dup	p.(Ser113Phefs*55)	1	NA	6617669dup	R. Williams, personal communication
Exon 04	c.357dup	p.(Leu120Serfs*18)	1	NA	6617649dup	Zhong et al. (2000)
Exon 04	c.379 C>T	p.(Arg127*)	2	Pathogenic	6617627 G>A	Sleat et al. (1999)
Exon 04	c.380 G>A	p.(Arg127Gln)	10	Pathogenic	6617626 C>T	Zhong et al. (2000)
Intron 04	c.380 + 55 G>A	NA	1	NA	6617571 C>T	Mole et al. (2001)
Intron 04	c.381–17_381–4del	NA	1	NA	6617432_6617445del	Chang et al. (2012)
Intron 04	c.381–2 A>G	NA	1	Likely pathogenic	6617430 T>C	Zhong et al. (2000)
Intron 04	c.381–1 G>C	NA	6	NA	6617429 C>G	Kousi et al. (2012)
Exon 04	c.377_387del	NA	2	NA	6617422_6617629del	Sleat et al. (1999)
Exon 05	c.406_409dup	p.(Glu139Glyfs*1)	1	NA	6617400_6617403dup	Chang et al. (2012)
Exon 05	c.457 T>C	p.(Ser153Pro)	1	NA	6617352 A>G	Caillaud, Manicom, Peuch, Lobel, and Poenaru (1999)
Exon 05	c.481 C>T	p.(Gln161*)	1	NA	6617328 G>A	R. Wang, personal communication
Exon 05	c.497dup	p.(His166Glnfs*22)	1	NA	6617312dup	Kousi et al. (2012)
Intron 05	c.509–1 G>A	NA^a^	5	Pathogenic	6617154 C>T	Sleat et al. (1999)
Intron 05	c.509–1 G>C	NA^b^	193	Pathogenic	6617154 C>G	Sleat et al. (1999)
Exon 06	c.605 C>T	p.(Pro202Leu)	1	Likely pathogenic	6617057 G>A	Mole et al. (2001)
Exon 06	c.616 C>T	p.(Arg206Cys)	8	Pathogenic	6617046 G>A	Berry‐Kravis et al. (2000)
Exon 06	c.617 G>A	p.(Arg206His)	1	Likely pathogenic	6617045 C>T	Kousi et al. (2012)
Exon 06	c.622 C>T	p.(Arg208*)	165	Pathogenic	6617040 G>A	Sleat et al. (1999)
Exon 06	c.625 T>C	p.(Tyr209His)	2	NA	6617037 A>G	(Kousi et al., 2012)
Exon 06	c.640 C>T	p.(Gln214*)	3	Pathogenic	6617022 G>A	(Kousi et al., 2012)
Exon 06	c.646 G>A	p.(Val216Met)	2	NA	6617016 C>T	(Wang et al., 2011)
Exon 06	c.650 G>T	p.(Gly217Asp)	1	NA	6617012 C>A	Chang et al. (2012)
Exon 07	c.713 C>G	p.(Ser238*)	1	NA	6616834 G>C	Kousi et al. (2012)
Exon 07	c.731 T>C	p.(Met244Thr)	1	NA	6616816 A>G	M. Nickel, personal communication
Exon 07	c.775del	p.(Arg259Valfs*17)	4	NA	6616772del	Goldberg‐Stern, Halevi, Marom, Straussberg, and Mimouni‐Bloch (2009)
Exon 07	c.790 C>T	p.(Gln264*)	4	NA	6616757 G>A	Kousi et al. (2012)
Exon 07	c.822_837del	p.(Leu275*)	1	NA	6616757 G>A	Kousi et al. (2012)
Exon 07	c.797 G>A	p.(Arg266Gln)	1	Uncertain	6616750 C>T	Kousi et al. (2012)
Exon 07	c.824 T>C	p.(Leu275Pro)	1	NA	6616723 A>G	Shen et al. (2013)
Exon 07	c.827 A>T	p.(Asp276Val)	14	Pathogenic	6616720 T>A	Kohan et al. (2009)
Exon 07	c.829 G>A	p.(Val277Met)	1	Not provided	6616718 C>T	Ju et al. (2002)
Exon 07	c.833 A>C	p.(Gln278Pro)	1	NA	6616714 T>G	Ju et al. (2002)
Exon 07	c.843 G>T	p.(Met281Ile)	1	NA	6616704 C>A	Kousi et al. (2012)
Exon 07	c.851 G>T	p.(Gly284Val)	35	Pathogenic	6616696 C>A	Zhong et al. (2000)
Exon 07	c.857 A>G	p.(Asn286Ser)	4	Pathogenic	6616690 T>C	Steinfeld et al. (2002)
Exon 07	c.860 T>A	p.(Ile287Asn)	1	Not provided	6616687 A>T	Sleat et al. (1999)
Intron 07	c.887–18 A>G	NA^c^	1	Likely pathogenic	6616521 T>C	Sleat et al. (1999)
Intron 07	c.887–10 A>G	varies	12	Conflicting	6616513 T>C	Noher de Halac et al. (2005)
Exon 08	c.887 G>A	p.(Gly296Asp)	2	NA	6616503 C>T	(Reid et al., 2016)
Exon 08	c.959 T>G	p.(Val320Gly)	1	Uncertain	6616431 A>C	E. de los Reyes, personal communication
Exon 08	c.972_979del	p.(Ser324Argfs)	3	Likely pathogenic	6616411_6616418del	Sleat et al. (1999)
Exon 08	c.984_986del	p.(Asp328del)	1	NA	6616404_6616406del	(Kousi et al., 2012)
Exon 08	c.987_989delinsCTC	p.(Glu329_Asp330delinsAspSer)	1	NA	6616401_6616403delinsGAG	Kousi et al. (2012)
Exon 08	c.1015 C>T	p.(Arg339Trp)	4	Pathogenic/likely pathogenic	6616375 G>A	Kousi et al. (2012)
Exon 08	c.1016 G>A	p.(Arg339Gln)	3	Conflicting	6616374 C>T	Kousi et al. (2012)
Exon 08	c.1027 G>A	p.(Glu343Lys)	3	Not provided	6616363 C>G	Sleat et al. (1999)
Exon 08	c.1029 G>C	p.(Glu343Asp)	1	Pathogenic	6616361 C>G	Dy et al. (2015)
Exon 08	c.1048 C>T	p.(Arg350Trp)	3	NA	6616342 G>A	R. Williams, personal communication
Exon 07	c.1049 G>A	p.(Arg350Gln)	1	Uncertain	6616341 C>T	M. Nickel, personal communication
Exon 08	c.1052 G>T	p.(Gly351Val)	2	NA	6616338 C>A	personal communication from relative
Exon 08	c.1057 A>C	p.(Thr353Pro)	2	Not provided	6616333 T>G	Steinfeld et al. (2002)
Exon 08	c.1058 C>A	p.(Thr353Asn)	1	Likely pathogenic	6616332 G>T	R. Wang, personal communication
Exon 08	c.1062del	p.(Leu355Serfs*72)	1	NA	6616328del	(Kousi et al., 2012)
Exon 08	c.1064 T>C	p.(Leu355Pro)	1	NA	6616326 A>G	Kousi et al. (2012)
Exon 08	c.888_1066del	p.His298Leufs*3	1	NA	6616324_6616502del	Kousi et al. (2012)
Intron 08	c.1075 + 2 T>C	NA	1	NA	6616313 A>G	Kousi et al. (2012)
Intron 08	c.1075 + 2 T>G	NA	1	NA	6616313 A>C	Sleat et al. (1999)
Intron 08	c.1076–2 A>G	NA	1	NA	6616076 T>C	Caillaud et al. (1999)
Intron 08	c.1076–2 A>T	NA	3	NA	6616076 T>A	A. Simonati, personal communication
Exon 09	c.1094 G>A	p.(Cys365Tyr)	6	Pathogenic/likely pathogenic	6616057 C>T	Sleat et al. (1999)
Exon 09	c.1093 T>C	p.(Cys365Arg)	1	pathogenic	6616057 A>G	Sleat et al. (1999)
Exon 09	c.1107_1108del	p.(Gly370Lysfs*32)^d^	1	NA	6616042_6616043del	Kohan et al. (2013)
Exon 10	c.1146 C>G	p.(Ser382Arg)	1	NA	6615562 G>C	Kousi et al. (2012)
Exon 10	c.1154 T>A	p.(Val385Asp)	1	Not provided	6615554 A>T	Sleat et al. (1999)
Exon 10	c.1166 G>A	p.(Gly389Glu)	4	Pathogenic	6615542 C>T	Sleat et al. (1999)
Exon 10	c.1204 G>T	p.(Glu402*)	2	NA	6615504 C>A	Kousi et al. (2012)
Exon 10	c.1226 G>T	p.(Gly409Val)	1	NA	6615482 C>A	C. Fagerstrom, personal communication
Exon 10	c.1239_1240ins6	p.(Ser413_Asn414ins2)	1	NA	6615468_6615469ins6	R. Williams, personal communication
Exon 10	c.1261 T>A	p.(Tyr421Asn)	1	NA	6615447 A>T	M. Nickel, personal communication
Exon 10	c.1266 G>C	p.(Gln422His)	11	Pathogenic	6615442 C>G	(Sleat et al., 1999)
Intron 10	c.1266 + 5 G>A	NA	1	Conflicting	6615437 C>T	Sleat et al. (1999)
Exon 11	C.1278 A>B	p.(Val426Val)	2	NA	6615318 A>B	Noher de Halac et al. (2005)
Exon 11	c.1284 G>T	p.(Lys428Asn)	1	NA	6615312 C>A	Ju et al. (2002)
Exon 11	c.1340 G>A	p.(Arg447His)	8	Pathogenic	6615256 C>T	Sleat et al. (1999)
Exon 11	c.1343 C>T	p.(Ala448Val)	1	NA	6615253 G>A	Kousi et al. (2012)
Exon 11	c.1351 G>T	p.(Asp451Tyr)	2	NA	6615245 C>A	R. Wang, personal communication
Exon 11	c.1358 C>A	p.(Ala453Asp)	2	NA	6615238 G>T	Kohan et al. (2013)
Exon 11	c.1358 C>T	p.(Ala453Val)	2	NA	6615238 G>A	Kohan et al. (2009)
Exon 11	c.1361 C>A	p.(Ala454Glu)	1	Not provided	6615235 G>T	Sleat et al. (1999)
Exon 11	c.1376 A>C	p.(Tyr459Ser)	4	Likely pathogenic	6615220 T>G	(Bhavsar et al., 2016)
Exon 11	c.1379 G>A	p.(Trp460*)	1	Pathogenic/likely pathogenic	6615217 C>T	Zhong et al. (2000)
Exon 11	c.1397 T>G	p.(Val466Gly)	6	Pathogenic	6615199 A>C	Sun et al. (2013)
Exon 11	c.1417 G>A	p.(Gly473Arg)	2	Not provided	6615179 C>T	Lam et al. (2001)
Exon 11	c.1424 C>T	p.(Ser475Leu)	9	Not provided	6615172 G>A	Sleat et al. (1999)
Exon 11	c.1424del	p.(Ser475Trpfs*13)	8	NA	6615172del	Moore et al. (2008)
Intron 11	c.1425 + 1 G>C	NA	1	NA	6615170 C>G	Kousi et al. (2012)
Exon 12	c.1439 T>G	p.(Val480Gly)	2	NA	6614978 A>C	Elleder et al. (2008)
Exon 12	c.1442 T>G	p.(Phe481Cys)	1	NA	6614975 A>C	Ju et al. (2002)
Exon 12	c.1444 G>C	p.(Gly482Arg)	2	Not provided	6614973 C>G	Kousi et al. (2012)
Exon 12	c.1467del	p.(Asn489Lysfs*29)	1	NA	6614950del	R. Williams, personal communication
Exon 12	c.1497del	p.(Gly501Alafs*18)	1	Pathogenic	6614920del	Kousi et al. (2012)
Exon 12	c.1501 G>T	p.(Gly501Cys)	1	NA	6614916 C>A	Kousi et al. (2012)
Exon 12	c.1510 A>T	p.(Asn504Tyr)	1	NA	6614907 T>A	Kousi et al. (2012)
Exon 12	c.1525 C>T	p.(Gln509*)	20	NA	6614892 G>A	Caillaud et al. (1999)
exon 12	c.1547_1548insTCAT	p.(Asp517Hisfs*1)	1	NA	6614869_6614870insATGA	Chang et al. (2012)
Exon 12	c.1547_1548del	p.(Phe516*)	3	NA	6614869_6614870del	Kousi et al. (2012)
Intron 12	c.1551 + 1 G>A	NA	1	Likely pathogenic	6614865 C>T	Wang et al. (2011)
intron 12	c.1551 + 1 G>T	NA	1	NA	6614865 C>A	Yu, Liu, Chen, Zhang, and Wang (2015)
Intron 12	c.1551 + 5_1551 + 6delinsTA	NA	3	NA	6614860_6614861delinsTA	Kousi et al. (2012)
Intron 12	c.1552–1 G>C	NA	2	NA	6614687 C>G	Sleat et al. (1999)
Exon 13	c.1595dup	p.(Gln534Profs*74)	1	NA	6614643dup	Sleat et al. (1999)
Exon 13	c.1603 G>C	p.(Gly535Arg)	2	NA	6614635 C>G	Kohan et al. (2013)
Exon 13	c.1613C>A	p.(Ser538Tyr)	1	NA	6614625 G>T	Yu et al. (2015)
Exon 13	c.1611_1621del	p.(Cys537Trpfs*67)	1	NA	6614617_6614627del	Caillaud et al. (1999)
Exon 13	c.1626 G>A	p.(Trp542*)	1	NA	6614612 C>T	C. Fagerstrom, personal communication
Exon 13	c.1630C>T	p.(Pro544Ser)^e^	1	Not provided	6614608 G>A	Zhong et al. (2000)
Exon 13	c.1642T>C	p.(Trp548Arg)	1	NA	6614596 A>G	Zhong et al. (2000)
Exon 13	c.1644 G>A	p.(Trp548*)	1	NA	6614594 C>T	Kousi et al. (2012)
Exon 13	c.1678_1679del	p.(Leu560Thrfs*47)	4	NA	6614559_6614560del	Sleat et al. (1999)
Exon 07	NA	Uncharacterised 1‐bp deletion	1		NA	Ju et al. (2002)
	Uncharacterised 5′ rearrangement resulting in insertion of intron sequences and frameshift	NA	1	NA	NA	Hartikainen et al. (1999)

^a^ Previously described as splice defect / p.(Gly171Thrfs*5).

^b^ Previously described as p.(Phe169*).

^c^ Previously described as splice defect / frameshift / deletion insertion.

^d^ Predicted amino acid effect using EMBOSS.

^e^ Previously described as p.(Ala555Pro).

NA, not applicable.

The variant c.299 A>G (p.(Gln100Arg)) has a frequency below 5% (National Center for Biotechnology Information, 2018) and is predicted benign. It occurred as an additional allele in three unrelated patients with classic late‐infantile CLN2 disease (Sleat et al., 1999; Tessa, Simonati, Tavoni, Bertini, & Santorelli, 2000), but it also occurred as the disease‐associated allele in trans with c.1266 + 5 G>A in a patient from Canada (disease phenotype unknown) (Kousi et al., 2012). The underlying sequence change for variant p.(Val426Val) is not available, therefore this variant cannot be assessed for potential alteration to splicing. In our data set, this variant occurred in two unrelated patients from Argentina, together with another variant of uncertain significance (c.89 + 4 A>G; Kohan et al., 2013; Noher de Halac et al., 2005). This latter variant potentially causes alteration of splicing and has been described as disease‐associated in one patient from Argentina (disease phenotype unknown). Finally, c.1501 G>T (p.(Gly501Cys)) is predicted as probably damaging and occurs in one patient from Turkey (disease phenotype unknown) (Kousi et al., 2012). It occurs (phase unknown) with c.622 C>T (p.(Arg208*)) and c.1343 C>T (p.Ala448Val)), which is also predicted as probably damaging. For the latter three additional alleles, there is no information on population frequency. Thus, for these four cases, the assignment of disease‐association is equivocal. Other additional alleles excluded from the analyses were listed in ClinVar as benign and/or have a prevalence in the population>5%. To date, of the 131 variants reported in the UCL database as disease‐associated, only 39 (30%) are recorded in ClinVar with an associated clinical classification.

**Figure 1:**
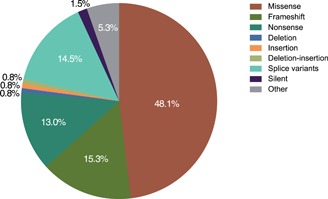
Spectrum of TPP1 variants described

**Figure 2:**
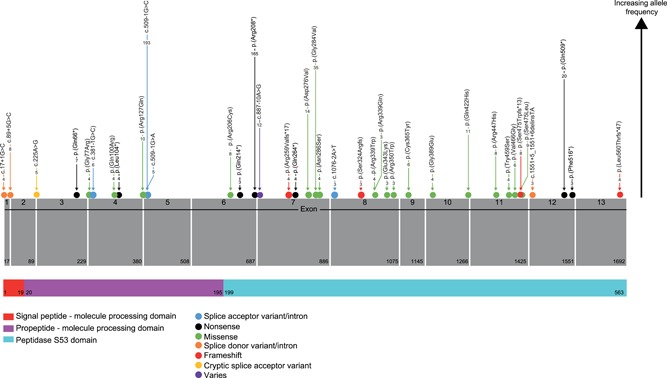
TPP1 gene structure and variants reported>two times. Domain information from InterPro accession O14773. Numbers within the arrows are the frequency with which variants were reported in the registry

Of the variants where relatedness could be established, 86/131 disease‐associated variants (66%) were private to single families. The spectrum of disease‐associated variants (131) was dominated by missense variants (63, 48%) followed by frameshift (21, 16% each) and nonsense (17, 13%) variant classes (Figure 1). Disease‐causing variants appear along the length of the *TPP1* gene, including the propeptide domain (Figure 2).

### 3.2 Genotype–phenotype correlation

Clinical phenotype classification was available in 65% of reports (254/389). Of those with reported phenotype classifications, the majority were classic late‐infantile (87%), with 13% atypical CLN2 disease (8% juvenile, 3% spinocerebellar ataxia or SCAR7, and <1% spastic paraplegia or congenital disease). Note that NCL phenotype descriptions were based on the age of disease onset: congenital, around birth; infantile, 0.5–1.5 years; late‐infantile, 2–4 years; juvenile, 5–10 years. Most individuals (87% [337/389]) had both alleles identified; of these, homozygosity was reported in 151 (45%) patients.

Geographical information was available for 356 individuals (92% of cases), with the majority of patients originating from Europe (217) followed by North America (78), South America (28), Asia (9), the Middle East (9), a mixture of Europe and other countries (8), Central America (5), and Africa (2).

**Figure 3:**
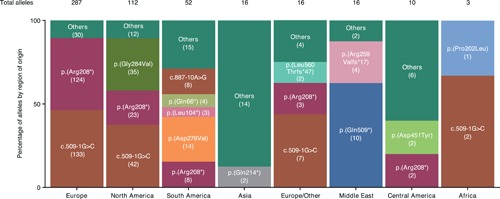
Most common alleles listed in the TPP1 locus‐specific database by region of origin. The number of times an allele was encountered is shown in parentheses. North America includes Newfoundland. Note: The emphasis now is on collecting new variants; frequency of the most common variants is, therefore, underrepresented here as new reports for these are no longer included in the UCL TPP1 locus‐specific database.

Overall, the two most frequently reported variants (c.509–1 G>C and c.622 C>T [p.(Arg208*)]) collectively occurred in 60% of individuals reported and accounted for 50% of disease‐associated alleles (Table 2). On a regional level, these variants appeared less frequently outside Europe and North America (Figure 3). The allele c.851 G>T (p.(Gly284Val)), originally identified in Newfoundland, was the second most common allele in North America and is a well‐characterized founder effect mutation in classic late‐infantile CLN2 disease (Fietz et al., 2016). In addition, c.1525 C>T (p.(Gln509*)) was the predominant mutated allele reported in the Middle East and also occurred in Europe. So far, c.640 C>T (p.(Gln214*)) has only been reported in China and Italy; these may also be examples of founder mutations.

**Table 2:** Disease‐associated *TPP1* variants reported ≥ 10 times

Nucleotide change	Amino acid change	Number of times reported (% of reported alleles, *N* = 717)
c.509–1 G>C	Splice acceptor variant	193 (27%)
c.622 C>T	p.(Arg208*)	165 (23%)
c.851 G>T	p.(Gly284Val)	35 (4.9%)
c.1525 C>T	p.(Gln509*)	20 (2.8%)
c.827 A>T	p.(Asp276Val)	14 (2.0%)
c.887–10 A>G	Variable	12 (1.7%)
c.1266 G>C	p.(Gln422His)	11 (1.5%)
c.380 G>A	p.(Arg127Gln)	10 (1.4%)

*Note*: Nucleotide changes are according to NM_000391.3; protein changes are according to NP_000382.3. The emphasis now is on collecting new variants; frequency of the most common variants is, therefore, underrepresented here as new reports for these are no longer included in the UCL *TPP1* locus‐specific database.

Disease alleles occur either as homozygous or as compound heterozygous, and the disease phenotype manifested likely reflects the combined effect of the alleles on TPP1 function. The NCL database presents data from individual patients, allowing supposition of genotype‐phenotype correlation (https://www.ucl.ac.uk/ncl-disease/)

Several variants have only been associated with the classic late‐infantile CLN2 disease phenotype. The c.851 G>T (p.(Gly284Val)) variant appeared 27 times in the collected population, in individuals with classic late‐infantile CLN2 disease from 26 patients in Canada and one patient in the US. Similarly, c.827 A>T (p.(Asp276Val)) was reported only in Argentina and Chile. The c.616 C>T (p.Arg206Cys) variant was also unique to classic late‐infantile CLN2 disease, and three out of the four patients reported to have it resided in India.

One large deletion, spanning exon 8, was reported (c.888_1066del [p.(His298Leufs*3)]) in one patient from the US with unrecorded phenotype. Substitutions at residue 343 are reported in patients with different phenotypes: p.(Glu343Lys); c.1027 G>A in classic late‐infantile CLN2 disease and p.(Glu343Asp); c.1027 G>A in atypical CLN2 disease. A number of variants were only associated with atypical CLN2 disease. The c.887–10 A>G (variable amino acid change) variant, which was reported 12 times and only in South America (Argentina, Chile, Colombia), Portugal, and Spain, likely causes an in‐frame inclusion of intron 7 and appears mainly in patients with a juvenile age of onset. Lastly, c.1397 T>G (p.(Val466Gly)) has only been reported six times in patients from the Netherlands with atypical CLN2 disease (SCAR7), in trans with the common c.509–1 G>C splice variant.

### 3.3 Biological significance

**Figure 4:**
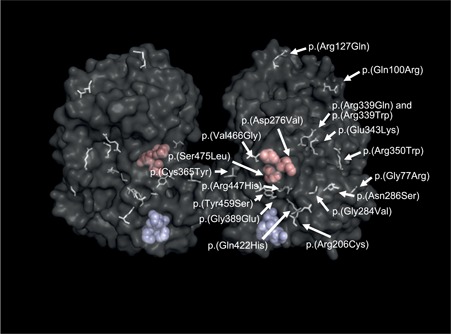
TPP1 proenzyme structure and missense variants reported ≥ three times. Three‐dimensional structure of TPP1 dimers (Pal et al., 2009). Active site (catalytic triad, Ser475‐Glu272‐Asp360) pocket residues are shown as red space‐filling models, calcium binding sites (Asp517‐Val518‐Gly539‐Asp543) in blue.

*TPP1* (NM_000391.3) maps to chromosome 11p15 and encodes the lysosomal exopeptidase, TPP1. Upon acidification, the inactive proenzyme form (Figure 4) is processed to a 46 kDa protein. The mature enzyme cleaves tripeptides from the amino terminus of small polypeptides undergoing degradation in the lysosomes and has weak endopeptidase activity (Lin, Sohar, Lackland, & Lobel, 2001; Pal et al., 2009). In vivo substrates of TPP1 are not well characterized and the pathological mechanisms underlying the disease remain unclear (Cooper, Tarczyluk, & Nelvagal, 2015; Palmer, Barry, Tyynelä, & Cooper, 2013; Stumpf et al., 2017).

Mutations identified in *TPP1* are distributed over the whole protein structure (Figure 2) and the majority are likely linked to loss of enzyme activity, though very few will have been confirmed to do this biochemically. Loss of TPP1 activity leads to neuropeptide degradation failure and significant accumulation of subunit c of ATP synthase. However, accumulation of subunit c has been identified in most forms of NCL and other lysosomal storage disorders, suggesting that this may not be the primary metabolic error in TPP1 deficiency (Palmer et al., 2013; Ryazantsev, Yu, Zhao, Neufeld, & Ohmi, 2007). Several common pathogenic cascades have been identified in lysosomal storage disorders, including altered lipid trafficking, autophagy, altered calcium homeostasis and oxidative stress (Vitner, Platt, & Futerman, 2010). Specifically, in vitro studies have linked TPP1 deficiency to oxidative stress and changes in mitochondrial morphology (Van Beersel et al., 2013). Regardless of the initiating mechanisms, the uniform neuropathological features of the NCLs may suggest the existence of shared pathogenic pathways for NCL proteins (Haltia, 2006; Palmer et al., 2013).

### 3.4 Clinical and diagnostic relevance

Diagnosis of CLN2 disease may be reached through a mixture of clinical findings, TPP1 enzyme deficiency, and/or molecular findings in *TPP1* (Fietz et al., 2016). Historically, diagnoses of NCL subtypes have relied on histopathological techniques, such as an electron microscope evaluation of autofluorescent storage material morphology, together with a clinical review of disease onset and symptoms (Williams et al., 2006). Assaying of white blood cell TPP1 activity is now the mainstay of diagnosis for TPP1*‐*related diseases (Fietz et al., 2016). Whereas this provides a direct test for CLN2 disease, it requires a specific suspicion of CLN2 or other NCL. By that point, there will have been significant disease progression and neurodegeneration (Nickel et al., 2016).

Alongside the demonstration of deficient TPP1 enzyme activity, detection of two pathogenic mutations in trans is considered the gold standard for CLN2 disease diagnosis (Fietz et al., 2016). Unlike biochemical testing, molecular genetic testing can be used to test multiple etiologies, and potentially lead to a patients phenotype. This means that no specific suspicion of an etiology is required, positioning these broad next‐generation sequencing (NGS)‐based tests as a tool for earlier diagnosis of genetic diseases. NGS techniques such as whole exome sequencing (WES) have emerged in recent years as useful tools for enhancing NCL subtype classification, particularly when mutations in different genes cause similar and overlapping phenotypes (Patiño et al., 2014). Timely diagnosis facilitates the early initiation of appropriate disease‐specific care and enables families to make informed decisions about treatment goals (Williams et al., 2017). Unexplained seizures, especially if preceded by early language developmental delay, can be an early symptom of CLN2 disease. In children without a specific CLN2 indication who present with delayed language skills, experts recommend investigating pediatric‐onset seizures using an epilepsy gene panel (Fietz et al., 2016; Lemke et al., 2012), as an approach to decrease time to the differential diagnosis of CLN2 disease.

Patients with CLN2 may encounter diagnostic delay due to the inexperience of their treating physicians and lack of awareness of NCL disorders. This may be a particular challenge in countries with an abundance of variant phenotypes due to diverse ethnic populations (Kohan et al., 2009). In an era where broad molecular tests are being used (e.g., gene panels, WES), the professional interpreting and/or conveying of test results to physicians, patients, or families is likely to not be an expert in CLN2 or NCLs overall. Experts in the area may know where to easily locate the *TPP1* locus‐specific database at UCL, but this database is less well‐known by general physicians. Central databases, like ClinVar, are widely used for all genetic diseases and are positioned as resources for any medical professional. The sharing of variant and clinical information with both NCL/CLN2 expert audiences, as well as non‐experts, facilitates both efficient researches of CLN2 disease and other NCLs and accurate interpretation of genetic testing results.

Interpreting the variants identified by molecular genetic tests can be cumbersome or unclear, particularly in cases of novel missense and/or in frame variation. In CLN2 disease, most patients (60%, in this database) have one of two common variants (c.509–1 G>C and c.622 C>T [p.(Arg208*)]), which have been consistently reported as pathogenic. If patients have any indication of CLN2 disease, and molecular testing finds any pathogenic or likely pathogenic variant in *TPP1*, TPP1 enzyme activity testing can be used to confirm the diagnosis. In addition, if a second variant is not identified, but enzyme activity is deficient, this can be used as evidence to classify any other potentially deleterious variants in the patient as well as provide a laboratory‐based diagnosis of CLN2 disease (Fietz et al., 2016; Richards et al., 2015).

### 3.5 Relevant animal models

The *TPP1* gene shows wide conservation in vertebrates (e.g., human, macaque, mouse, and cow; Wlodawer et al., 2003), but there is no obvious orthologue in lower organisms (e.g., *Drosophila*, *Caenorhabditis elegans*, or *Saccharomyces cerevisiae*). However, TPP1 does belong to a family of enzymes with members found in bacteria (Oda et al., 1996; Oda, Takahashi, Tokuda, Shibano, & Takahashi, 1994). A novel TPP1 orthologue, located in the lysosomes of the amoeba *Dictyostelium discoideum*, has also been described (Phillips & Gomer, 2015).

Zebrafish with a TPP1 deficiency die prematurely and show ubiquitous storage material containing ATP synthase subunit c, with it being more evident in the CNS and muscles (Mahmood et al., 2013). The early stop codon in exon 3 (which is also described in humans but at a different amino acid position) leads to an early‐onset neurodegenerative phenotype and functional motor impairment preceded by a phase of hyperactivity that could be consistent with seizures. The zebrafish model also shows significant apoptotic cell death and aberrant proliferation in the optic tectum, cerebellum, and retina. As mouse models for CLN2 disease do not seem to suffer from visual problems or retinal degeneration (see below), the study of this aspect of the disease could utilize these findings from the zebrafish model.

The first TPP1 deficient mouse model (mixed background [C57BL/6:129S6]) was generated by knock‐in of the CLN2‐specific p.(Arg447His) missense mutation into the *Tpp1* gene in combination with a large intronic insertion (Sleat et al., 2004). The lack of activity of TPP1 protein does not affect the initial stages of development but evolves with signs of progressive neurological deficits with aging. The lifespan is drastically reduced (median survival 138d) and the mice display early motor deficits, seizures, spontaneous tremors, and ataxia (Sleat et al., 2004). The neurological impairment is visible in the brain, spinal cord, and peripheral sensory neurons, with an accumulation of autofluorescent material in the lysosomes. The severe loss of neurons in the cerebral cortex that is observed in the human late‐infantile CLN2 disease is not that obvious, but there is a clear loss of Purkinje cells which could be linked to the cerebellar ataxia. Studies on the histology of the retina do not show any loss of photoreceptors or any reduction in cell layers (Sleat et al., 2004).

More recently, a mouse model encoding the most common nonsense mutation found in humans, an early stop codon instead of arginine in the 208 positions (p.Arg207* in mice), has been generated and described (Geraets et al., 2017). The resulting transcript reduction leads to reduced enzymatic activity in different organs, such as the liver, spleen, or cerebellum. Consequently, mice show a reduced lifespan, with most dying between 3 and 6 months of age. As observed in the previous mouse model (Sleat et al., 2004), impaired motor behavior is observed and characterized by tremors, seizures, hyperactivity, and strength deficits. The visual phenotype was not studied. Histological evaluation of the brain displayed an accumulation of the mitochondrial ATP synthase subunit c in superficial or deep cortical layers. Showing a similar phenotype to the pre‐existing mouse model, this new transgenic mouse could be used for the preclinical evaluation of all therapeutic approaches including mutation‐guided therapies.

To date there is evidence of NCL in over 20 canine breeds and mixed‐breed dogs (Katz, Rustad et al., 2017). The canine *Tpp1* gene sequence (GenBank AF114167) includes all 13 exons that are present in the human *TPP1* gene, and exonic sequences are highly conserved between both species (Drögemüller, Wöhlke, & Distl, 2005).

The first report of NCL in Dachshund dogs described a neurodegenerative disease starting with hind‐leg weakness at 3 years of age (Cummings & de Lahunta, 1977). Histological analysis showed cerebellar atrophy together with marked loss of neurons and Purkinje cells in the area. Ultrastructural studies revealed various membrane‐bound inclusions in addition to the autofluorescent lipofuscin granules. No genetics or biochemical analysis was performed in this study, so the genetic basis of this disease is unknown. However, another Dachshund dog model, with earlier disease onset, was found to be homozygous for the mutant c.325delC allele in *Tpp1* (Awano et al., 2006). The first symptoms (vomiting, mental dullness, loss of housebreaking, and unresponsiveness to previously learned commands) were visible at approximately 9 months of age. The disease progressed with gradual loss of sight and ataxia (10 months), and myoclonus of the head with seizures (11 months). This model showed episodes of hyperactivity and howling, and later exhibited aggressive behavior, a hypermetric gait, and incessant circling. Vomiting became more frequent and diarrhea subsequently developed. Finally, diarrhea progressed to hematochezia, with death by 12 months of age. The TPP1 activity measured in the brain was less than 1% of that observed in the cortex of control dogs, resulting in autofluorescent storage bodies in all examined regions of the CNS. The ultrastructural electron microscopy analysis of the bodies consistently showed the curvilinear forms characteristic of the human CLN2 mutations (Awano et al., 2006).

All previously described animal models, regardless of the type of mutation, share the most common symptoms observed for human pathology due to the reduced levels of TPP1 protein. Thus, animal models are an invaluable resource to test different therapeutic strategies, such as gene therapy, cell transplantation, chemical compounds, or enzymatic replacement. Given the nature of the symptoms and the progression of the disease, most efforts are focused on the CNS and loss of vision. However, it is crucial to take into account that the storage bodies are found to accumulate in all visceral organs, so there may be extraneuronal pathology. A recent publication showed systemic signs of the disease in dogs after delayed neurological progression due to successful intracerebroventricular gene therapy (Katz et al., 2014, 2017; Vuillemenot et al., 2015). If the therapeutic approaches to treat the CNS succeed, animal models could provide valuable insight into further challenges affecting the life expectancy and the quality of life of the patients.

### 3.6 Future prospects

The newly approved intracerebroventricular enzyme replacement therapy has rendered CLN2 disease from an untreatable to treatable disease, especially if treatment is started early before significant neurodegeneration has already taken place (Schulz et al., 2018). The ultimate effort to improve early diagnosis of a now treatable disease is newborn screening (NBS). Experts suggest that assaying TPP1 activity with enzyme substrates compatible with tandem mass spectrometry detection could support future large‐scale NBS programs (Barcenas et al., 2014; Fietz et al., 2016). The adoption of successful NBS programs for CLN2 also relies heavily on the clarity of genotype–phenotype correlations. There must be a concerted effort to ascertain the disease liability of *TPP1* variants to facilitate interpretation of variants detected through population‐based screening and diagnostic molecular genetic testing. The algorithm for detection should maximize specificity, achieve a high positive predictive value, and have a low false‐positive rate (Pitt, 2010). There is also the more distant possibility of whole exome or whole genomic sequencing on all newborns, which could be followed by specific testing for predicted enzyme deficiencies. In addition, a robust understanding of genotype–phenotype correlations would facilitate interpretation of NBS data.

## 4 CONCLUSION

To date, 131 *TPP1* gene variants have been reported in 389 individuals with TPP1 deficiency. The majority of disease‐causing *TPP1* variants are private. Currently, only 30% of *TPP1* variants reported here are in the NCBI ClinVar database with an associated ACMG clinical classification. Most individuals with TPP1 deficiency have one of two variants: c.622 C>T (p.(Arg208*) or c.509–1 G>C. The uniform and timely reporting of all variants not only benefits families by providing a definitive diagnosis, but has also allowed genotype–phenotype correlations to be considered, and in some cases, reassessed. The inclusion of all variants in a database, disease‐causing or not, is useful. If a variant has previously been proved benign, this could expedite interpretation and diagnosis. Comprehensive reporting and data sharing is essential as molecular genetic testing increases as a first‐line diagnostic test for pediatric‐onset neurological disease. The long‐established NCL mutation database remains a valuable resource for collecting *TPP1* variants.

To contribute to the UCL NCL database, please contact Sara Mole (s.mole@ucl.ac.uk).

## CONFLICTS OF INTEREST

A. Schulz has received personal fees from BioMarin Pharmaceutical Inc., outside of the submitted work. M. Aristorena has no conflicts of interest to declare. M. Bailey was an employee of BioMarin Pharmaceutical Inc. at the time of the study. N. Miller was an employee of BioMarin Pharmaceutical Inc. at the time of the study. Professor S. E. Mole receives financial support from BioMarin Pharmaceutical Inc. to maintain the NCL Mutation Database and acts as an advisor to BioMarin Pharmaceutical Inc. on mutations in *TPP1*.
